# Combined Virtual and Experimental Screening for CK1 Inhibitors Identifies a Modulator of p53 and Reveals Important Aspects of in Silico Screening Performance

**DOI:** 10.3390/ijms18102102

**Published:** 2017-10-06

**Authors:** Vassilios Myrianthopoulos, Olivier Lozach, Danae Zareifi, Leonidas Alexopoulos, Laurent Meijer, Vassilis G. Gorgoulis, Emmanuel Mikros

**Affiliations:** 1 Department of Pharmacy, University of Athens, Panepistimiopolis Zografou, GR-15771 Athens, Greece; 2 Protein Phosphorylation & Human Disease Group, Station Biologique, B. P. 74, CEDEX 29682 Roscoff, Bretagne, France; olivier.lozach@univ-brest.fr; 3 ProtATonce Ltd., 15343 Athens, Greece; danae.zar@gmail.com; 4 School of Mechanical Engineering, National Technical University of Athens, 15780 Athens, Greece; leo@mail.ntua.gr; 5 ManRos Therapeutics, Perharidy Research Center, Roscoff, 29680 Bretagne, France; meijer@manros-therapeutics.com; 6 Department of Histology-Embryology, School of Medicine, University of Athens, Mikras Asias 75, GR-11527 Athens, Greece; vgorg@med.uoa.gr; 7 Biomedical Research Foundation of the Academy of Athens, 11527 Athens, Greece; 8 Faculty Institute of Cancer Sciences, University of Manchester, Manchester Academic Health Science Centre, Manchester M13 9NT, UK; 9 “Athena” Research and Innovation Center, 15125 Athens, Greece

**Keywords:** casein kinase-1, NCI diversity set-II, structure-based screening, ligand-based screening, Glide, ROCS, enrichment calculation, p53 levels, compound collection enumeration, screening template

## Abstract

A compound collection of pronounced structural diversity was comprehensively screened for inhibitors of the DNA damage-related kinase CK1. The collection was evaluated in vitro. A potent and selective CK1 inhibitor was discovered and its capacity to modulate the endogenous levels of the CK1-regulated tumor suppressor p53 was demonstrated in cancer cell lines. Administration of 10 μM of the compound resulted in significant increase of p53 levels, reaching almost 2-fold in hepatocellular carcinoma cells. In parallel to experimental screening, two representative and orthogonal in silico screening methodologies were implemented for enabling the retrospective assessment of virtual screening performance on a case-specific basis. Results showed that both techniques performed at an acceptable and fairly comparable level, with a slight advantage of the structure-based over the ligand-based approach. However, both approaches demonstrated notable sensitivity upon parameters such as screening template choice and treatment of redundancy in the enumerated compound collection. An effort to combine insight derived by sequential implementation of the two methods afforded poor further improvement of screening performance. Overall, the presented assessment highlights the relation between improper use of enrichment metrics and misleading results, and demonstrates the inherent delicacy of in silico methods, emphasizing the challenging character of virtual screening protocol optimization.

## 1. Introduction

A considerable number of computational tools are currently available for screening compound collections for bioactive molecules in silico. These methodologies play a prominent role, especially in the early stages of drug discovery where the main research focus is on the identification of the most promising hits that will subsequently comprise versatile starting points for hit-to-lead optimization studies. In this aspect, virtual screening methodologies constitute nowadays an integral part of most drug discovery endeavors in contemporary science and their contribution in improving the success rate of such campaigns is indisputable [1]. On the other hand, being in essence theoretical models with different degrees of empirical parameterization, these methods need be treated with a critical thought and always alongside will experimental data that will provide the adequate validity of all steps of research in which their use is involved. To this respect, the detailed and systematic assessment of virtual screening performance is a research topic of key importance for either promoting rational use of the various available algorithms and for facilitating methodological advancements in the field. The number of efforts being made to assess in silico screening performance is significant, including both scientific articles and high-quality preassembled training or decoy sets such as the Database of Useful Decoys-Enhanced (DUD-E) [2,3,4,5,6].

Virtual screening methodologies are of particular importance in drug discovery projects targeting disease areas of difficult druggability, such as those related with protein–protein interactions (PPIs) [7,8]. Among the pathways of challenging druggability are those involved in DNA damage repair (DDR). The DDR mechanisms constitute a machinery of high complexity with utmost importance for preservation of genetic integrity in healthy cells. Perturbations in the functionality of the numerous elements related with DDR either directly or as regulatory partners can cause serious pathological responses [9]. Indeed, there are numerous studies demonstrating the strong correlation between defects in DDR mechanisms on one hand and serious diseases on the other [10,11,12]. Nonetheless, there are instances where such defects can be exploited in a therapeutic perspective by pursuing a synthetic lethality effect [13]. In terms of small molecule modulators, the various DDR-related proteins demonstrate a wide range of druggability. This is rather well anticipated due to the diverse roles such proteins hold as scaffolds of multimeric assemblies and mediators or facilitators of extensive protein–protein and protein–DNA interactions. About 38% of all DDR-related proteins with a known structure are predicted as being druggable [9]. Among the most druggable modules in DDR mechanisms are specific members of the protein kinase family such as ataxia-telangiectasia mutated (ATM) and ataxia telangiectasia and Rad3-related (ATR), checkpoint kinases 1 and 2 (Chk1 and Chk2), and the mitotic kinases Aurora B and polo-like kinase-1 (PLK1), as well as cyclin-dependent kinases 1 and 2 (CDK1 and CDK2), glycogen synthase kinase-3β (GSK-3β), and casein kinase 2 (CK2), all of which have been identified as potential targets [9], although some of these enzymes are considered less attractive due to their heavy involvement in different pathways unrelated to DDR.

Although CK2 has been suggested as a low priority target for DDR, this is not the case for its distant relative casein kinase 1 (CK1). The mammalian CK1 group is comprised by seven isoforms (α, β, γ1, γ2, γ3, δ, ε) which are involved in a number of key physiological functions such as chromosome segregation and circadian rhythm regulation, and in signaling pathways crucial for development, such as Wnt and Hedgehog [14]. Most importantly, numerous studies provide a wealth of evidence for the central role of CK1 in DNA damage-related signal transduction (see reviews [14,15]). To this respect, the different isoforms of CK1 are involved in regulating critical components of the cellular response in DNA damage such as the tumor suppressor p53, either directly or through mouse double minute 2 (MDM2) phosphorylation [16,17]. The interaction between CK1 and p53 in response to DNA damage provides an interesting field of research, as indirect stabilization of p53 through targeting of an upstream protein kinase might provide an alternative and more efficient route instead of the challenging MDM-related PPI inhibition (e.g., by nutlins) [18]. CK1 is furthermore involved in regulation of other key cellular functions mediated by cell division cycle 25 (Cdc25A) phosphatase, claspin, phosphatase and tensin homolog (PTEN), topoisomerase II-α, and the DNA methylation maintenance factor ubiquitin-like containing PHD and RING finger domains, 1 (UHRF1) [15], while it has been recently reported that CK1 contributes to genomic stability control through directly regulating the levels of Chk1 kinase that functions at the mitotic and DNA response checkpoints [19]. In terms of inhibitor development, CK1 represents a moderately explored kinase. Inhibitors demonstrating various degrees of selectivity towards CK1δ/ε or cross-reactivity towards CDKs and p38 include the isoquinoline CK1–7 [20], roscovitine derivatives CR8 and DRF053 [21], the imidazoles d4476 [22], TA01, and TA02 [23], the benzothiazole LH846 [24], the pyrazolopyrimidine PF4800567 [25], and the imidazole pyrimidinamine PF670462 [26].

As a continuation of our efforts to critically assess the efficiency of virtual screening tools in lead discovery case studies against emerging drug targets and to devise high-performance in silico screening protocols based on consensus approaches [27,28], we present here a tandem theoretical and experimental evaluation of a compound collection with pronounced structural diversity towards the DDR-related kinase CK1. The present study aims at two complementary objectives. The first aim is to create a comprehensive and self-consistent dataset of experimentally determined biological activities and subsequently utilize it as a tool for benchmarking two typical and orthogonal virtual screening methodologies. The second aim of the study is to discover original chemotypes with promising CK1 inhibitory activity and capabilities to sustain future hit-to-lead optimization studies. With respect to the first objective, we focus on the impact upon enrichment and overall screening performance of a series of parameters not always considered as important in screening campaigns. We perform benchmarking in screens based on each of the two in silico methods individually and then we assess whether an approach based on their sequential implementation can be utilized for achieving optimal screening performance. Finally, with respect to the second objective, we report one novel scaffold of high originality and a promising bioactivity profile in terms of CK1 inhibition and selectivity, and we demonstrate the inhibitor potential to modulate the levels of downstream to CK1 DDR-related tumor suppressor p53 in cancer cells.

## 2. Results and Discussion

### 2.1. The National Cancer Institute Developmental Therapeutics Program (NCI/DTP) Diversity Set-II Compound Collection

As mentioned, the dual objective of this study was to screen for novel inhibitors of CK1 and, subsequently, to utilize the derived screening dataset for enabling comprehensive evaluation of the performance of two representative methods for virtual screening. To facilitate precise quantification of the efficiency and overall performance of the studied in silico tools and furthermore to determine their dependence upon the variation of key setup parameters, an entire small molecule collection was experimentally evaluated for the inhibitory properties of contained compounds against CK1. The screened collection was the NCI Diversity Set-II that is comprised by 1364 freely available molecules provided by the Developmental Therapeutics Program (DTP) Open Repository of National Cancer Institute (NCI). As suggested by its name, the main criteria of compound selection among the complete NCI/DTP Repository are structural diversity and drug-likeness. The NCI Diversity Set-II comes as a pre-processed collection assembled on the basis of a chemoinformatic analysis of three-point pharmacophore points.

### 2.2. Complete In Vitro Evaluation

All compounds of the collection were assessed for their inhibitory activity towards CK1δ/ε and the most active molecules were subsequently evaluated in terms of selectivity against the related kinases CDK5, GSK3β, and dual specificity tyrosine-phosphorylation-regulated kinase 1A (DYRK1α). These three proteins are extensively explored in terms of inhibitor development and most importantly, they are considered as off-targets with respect to DDR mechanisms due to their heavy involvement in a multitude of pathways unrelated to DDR [9]. All compounds were evaluated at a single concentration of 10 μM using suitable kinase substrates in the presence of 15 μM (γ-^33^P)-labeled ATP and residual enzyme activities were determined (residual activity is the percentage of enzyme activity after treatment with compounds, whereas low residual activity corresponds to high kinase inhibition and vice versa). A relatively high threshold of 40% in the residual activity was selected to discriminate between active and inactive molecules. This threshold was selected for creating a more uniform distribution between actives and inactives, and thus enable derivation of more comprehensive metrics of screening performance and enrichment, with reasonable compromise in the inhibitory potencies of the identified hits in terms of their future optimization prospects.

A total number of 19 compounds afforded measurable CK1δ/ε inhibition within the aforementioned 40% activity threshold and were ranked as hits. However, only 4 compounds demonstrated considerable inhibition and are presented along with their entry codes and structures in Table 1. Such a result affords a relatively low hit rate of 0.29% for the studied enzyme. With respect to these top-ranked hits, the dihydroxyquinoline sulphonamide NSC45572 (**1**) was the most promising compound, showing the strongest inhibitory activity towards CK1δ/ε with a residual kinase activity of only 7%. The compound was highly selective as well, showing no inhibition towards the other studied kinases (the residual activity was between 77% and 94%). Similar selectivity was determined for the isoindolobenzimidazolone NSC252777 (**2**) that, however, showed lower inhibitory potency, affording a residual CK1δ/ε activity of 18%. Within the specified activity threshold, two less potent CK1δ/ε inhibitors were additionally identified, namely the pyrimidine carbonitrile NSC71866 (**3**) and the pyrrolopyrimidine NSC105827 or thiosangivamycin (**4**), with corresponding residual enzyme activities of 14% and 20%, respectively. Yet, those molecules were not selective, as **3** also demonstrated considerable inhibitory activity against CDK5 (residual activity of 25%) and **4** inhibited both CDK5 and DYRK1α (residual activities of 7% and 12%, respectively).

### 2.3. Virtual Screening Performance and Enrichment Metrics

Access to a comprehensive and original set of experimentally determined biological activities represents an exciting opportunity for performing a systematic assessment of the performance of virtual screening methodologies. Having generated such a self-consistent and complete dataset, the next step of our study was the retrospective performance assessment of two representative and common virtual screening methods. Docking-scoring calculations represent the method of choice for virtual screening whenever the structure of the target protein is known. In this study, the Glide algorithm (Schrodinger Inc., New York City, NY, USA) was utilized for structure-based screening of the NCI Diversity Set-II inside the CK1-active site [29,30,31]. Glide is deemed as one of the most efficient virtual screening tools and, notably, it supports parameterization to a satisfactory extent by including adjustment of factors such as docking accuracy and simulation of induced-fit effects. On the other hand, in the absence of a suitable docking template the alternative ligand-based approach is usually pursued. The high-performance rapid overlay of chemical structures (ROCS) algorithm (OpenEye Inc., Santa Fe, NM, USA) was used for the ligand-based screening [32]. In this technique, a known active molecule is used as a three-dimensional query and the screened compounds are ranked based on their overall similarity with it. Similarity is quantified in terms of the overlap in real space of molecular shape and pharmacophoric sites such as hydrogen bond donors and acceptors, positive and negative atoms, hydrophobes, and aromatic rings between the query and unknown molecules.

Nevertheless, the detailed measurement and quantification of the efficacy of any in silico screening tool is not always a straightforward procedure [33,34,35,36]. It should be noted that both Glide and ROCS packages provide their own built-in enrichment calculators, which are comparable but not identical in content (in Glide there is the “Enrichment calculator” python-based script and in ROCS there is the “Perform validation run” option). In this study, a third-party tool was considered more appropriate for generating performance metrics, aiming at providing a common frame of reference for enrichment evaluation in a self-consistent and unbiased fashion. To this end, all related metrics presented hereafter were calculated using the Screening Explorer framework and the corresponding module available online [37]. The calculated metrics include the receiver operating characteristic (ROC) curve and the corresponding area under the curve (ROC AUC), the total gain (TG), the Boltzmann-enhanced discrimination of ROC (BEDROC), and the robust initial enhancement (RIE). The ROC curves account for the overall performance of a screening method in distinguishing between actives and inactives, the TG value quantifies the relation between variation of scores and detection of actives, and the BEDROC and RIE values account for the critical aspect of early recognition of actives in a specific screening dataset (for a detailed description and formalism of the metrics used please see [4,36]).

A specific issue in measuring virtual screening performance concerns the treatment of redundancy of screened compounds. In silico techniques demand that prior to screening, any collection of candidate molecules should be carefully preprocessed and enumerated in terms of the possible tautomeric and protonation states as well as stereoisomers of all contained entries. Given that most compound collections, including the NCI Diversity Set-II, are provided as two-dimensional structure files, conversion of the raw collection to a three-dimensional library inflates the library size by a factor that can vary from 4-fold or 10-fold in algorithms like Glide where conformer generation is integrated within the screening procedure, while the expansion can be as high as 1000-fold or more in algorithms where conformers are generated in advance of screening, such as in the case of ROCS. This expansion in library size is uneven, depends on the structural elements of each individual molecule (number of chiral centers, p*K*_a_, acidic protons or others), and can influence any measurement of screening performance by introducing a rather delicate inconsistency; either enrichment calculation should be based on the single best instance that each molecule (in a specific state) will appear as top-ranked in the screening results, or it should be based on the overall population of its various possible states (with respect to protonation, tautomerism, existence of stereoisomers, and even conformers in case such ensembles are pre-generated). Arguing for the first approach, one would say that only the state of each molecule that singly affords the optimal rank (in terms of docking or similarity scores) is of actual importance in each individual screening calculation. Regarding the second approach, the argument would be that given that the actual state of the screened molecules in the conditions of a specific experimental assay setting is in most cases practically not known, even for any true actives that might indeed be found, the most reasonable approach is to consider the distribution of all possible states for each molecule when comparing screening predictions with experiments for deriving enrichment measurements. Either approaches are implemented as built-in enrichment calculation modules in state-of-the-art screening software. In this study, both were followed in a comparative fashion and their effect on screening performance along with a critical consideration of their use is discussed in more detail below.

### 2.4. Structure-Based Screening

As discussed previously, Glide offers a reasonable degree of parameterization with two docking precision levels; the faster Glide-SP option emphasizing avoidance of false negatives, and the more time-demanding Glide-XP option focusing on a more detailed scoring scheme [31]. Moreover, while calculations are based on a rigid protein representation, the algorithm allows for a rough approximation of induced-fit effects by providing the option to scale down the Van der Waals radii of atoms, both in proteins or ligands, to a fraction of their nominal values. The optimal Van der Waals (VdW) scaling factors are usually characteristic of each target class, with the common fold dictating the overall degree of plasticity for homologous proteins. For fine-tuning Van der Waals scaling, the use of a training set encompassing congeneric compounds that span a wide range of binding affinities is generally recommended. In short, the procedure of fine-tuning consists of a small set of separate docking calculations utilizing the abovementioned training set and taking into account all possible combinations (in this case 9 combinations) for serially scaling both ligand and protein VdW radii by the factors of 0.8, 0.9, and 1 (no scaling). Subsequently, a linear regression of the experimental versus predicted free energies of binding is performed for each experiment, and optimal VdW scaling is selected on the basis of the docking calculation that produced the highest correlation. In this study, an extensive set of ATP-competitive kinase inhibitors previously published [38] by our group was used with kinase GSK-3β, and an optimal factor of 0.8 was determined for protein VdW scaling, indicating a non-negligible induced-fit effect which is in good accordance with the well-described plasticity of the kinase fold [39].

An important variable in virtual screening when following a rigid protein representation is selection of the optimal crystal structure to be used as a docking template. This parameter can be of key importance for screening performance, and although there is no standard procedure or any strict criteria for choosing the most appropriate structure, high resolution crystal structures where the studied protein is complexed with average-sized ligands demonstrating a “typical” binding geometry tend to be prioritized over apoenzyme structures or “atypical” interaction modes. Concerning kinases, for a screening campaign targeting ATP-competitive inhibitors, definition of a “typical” binding template would be that of a type-I or type-II inhibitor complex. Following the aforementioned reasonable yet arguably simplified approach, in the first step of this study we have selected a single high-quality co-crystal structure of human CK1γ featuring an ordinary ATP-competitive inhibitor at a resolution of 1.3 Å released by a structural genomics effort (pdb code: 2IZR).

**Figure 1:**
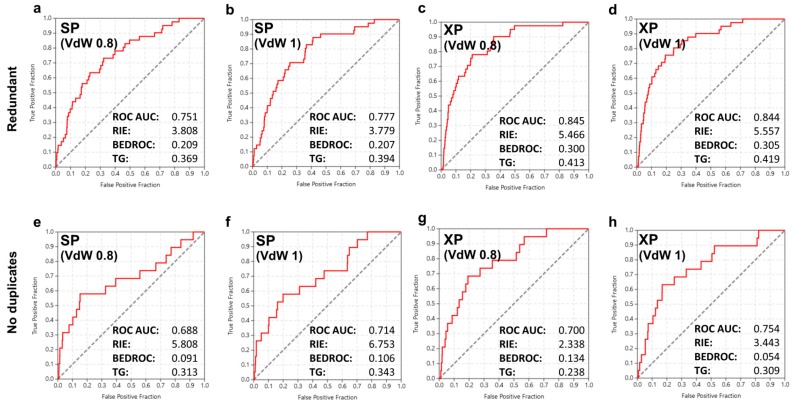
The receiver operating characteristic (ROC) curves and key enrichment metrics obtained for the initial structure-based screening of the NCI Diversity Set-II against CK1. In the first (**a**–**d**) and second (**e**–**h**) lines, data for screens following the two distinct approaches with respect to ranking of different possible states of screened compounds are depicted, as discussed in the text (redundancy treatment). The screening accuracy (SP or XP) and the Van der Waals scaling factors are shown inside the inlets. AUC: area under the curve; RIE: robust initial enhancement; BEDROC: Boltzmann-enhanced discrimination of ROC; TG: total gain.

Having prepared a docking template on the basis of a reasonable crystal structure and an optimal protein VdW setting, we probed for the effect on enrichment of three specific parameters considered as important with respect to screening performance. It should be noted that these parameters are largely unrelated to the individual docking template and thus they tend to have a more universal character. These parameters concern treatment of docking precision (SP or XP level of accuracy), redundancy (populations of possible ligand states or singe top-ranked states, discussed in the previous paragraphs and hereafter named “redundant” and “no duplicates” approaches, respectively), and ligand atom VdW radii scaling (no scaling or scaling down at 80% of nominal values). The screening results and corresponding enrichment graphs are summarized in Figure 1. All 8 structure-based screens showed a fairly good performance. However, enrichment metrics converged to the notion that the most critical parameter for screening performance was treatment of redundancy. A clear correlation between improvement of screening performance and treatment of redundancy was evident, as the ROC AUC scores were considerably increased in all docking experiments where the “redundant” approach was followed. The shift ranged between 0.05 and 0.15 and, given that the maximal reasonable variation of ROC AUC is 0.5 (0.5 indicates pure randomness and 1.0 an ideal performance), the influence of redundancy on screening performance was notably high, accounting for a variation between 10% and 30%. This trend was observed in all enrichment metrics, with the only discrepancy being the early enrichment as captured specifically by the RIE value that showed an opposite sign variation favoring the “no duplicates” approach in the screening results obtained at the SP docking precision level (from 3.808 to 5.808 and from 3.779 to 6.753, respectively; Figure 1a,e and Figure 1b,f). Yet in the same comparison, variation of the same factor of early enrichment, as captured this time by the alternative BEDROC metric, was in accordance with the general trend observed for total enrichment that favored the “redundant” over the “no duplicates” approach.

Concerning the effect of docking accuracy, the XP algorithm outperformed SP with respect to the overall screening performance. An improvement in ROC AUC ranging from 1.8% (from 0.688 to 0.700; Figure 1e,g) to 12.5% (from 0.751 to 0.845; Figure 1a,c) was consistently measured in the enrichment scores of screens performed at the XP accuracy level. However, the improvement was not dramatic and in terms of speed and accuracy balance, the use of the much slowed XP protocol might not be always justified. Another observation concerns early enrichment which was asymmetrically affected, with higher RIE and BEDROC scores results derived at the SP accuracy level in the “no duplicates” approach and a significant difference by approximately 50% observed on both metrics (from 5.808 to 2.338 and from 6.753 to 3.443, respectively; Figure 1e,h). A minor exception was obtained in the BEDROC score for the transition from VdW scaling of 0.8 toward no VdW scaling (from 0.091 to 0.134; Figure 1e,g). On the other hand, the overall effect of the ligand VdW scaling in screening performance was rather limited, showing a noticeable variation only to the “no duplicates” series of results, where no VdW scaling afforded a slightly better performance.

### 2.5. Ligand-Based Screening

As a second step, the performance of the ligand-based approach as implemented in ROCS was evaluated. Similarity-based algorithms are orthogonal to docking and usually offer more limited parameterization capacities compared to their structure-based counterparts. This characteristic underlines the decisive role the selected query structure can actually play to screening performance. As a means to address the impact of query selection and furthermore, to remove any possible bias arising from accidental similarities between the known actives and the NCI Diversity Set-II compounds, we selected 10 structurally distinct query molecules and performed a separate screen for each of those structures. All known actives were CK1 inhibitors co-crystallized with the target enzyme, so that their individual bioactive conformations were captured in the corresponding crystal structures (pdb codes: 2CHL, 2IZT, 3UZP, 4G17, 4HGS, 4HNF, 4KBA, 4TW9, 5IH5).

**Figure 2:**
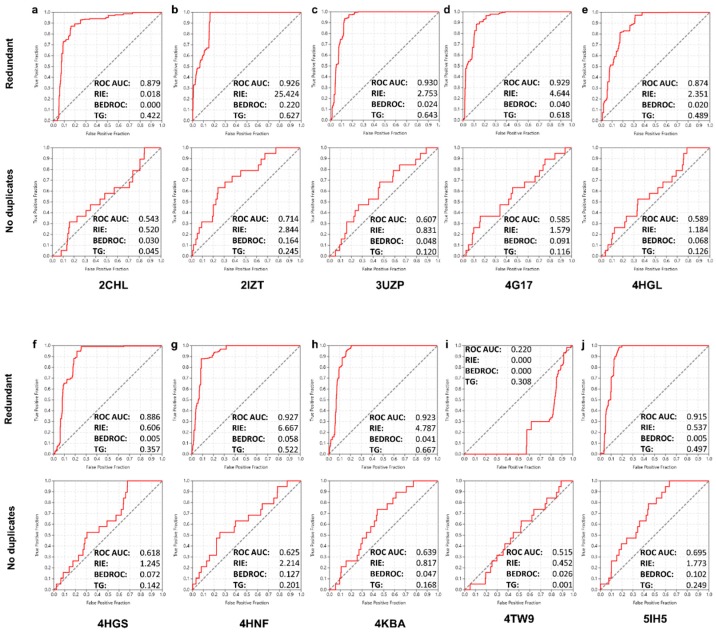
The ROC curves and key enrichment metrics obtained for the subsequent ligand-based screening of the NCI Diversity Set-II against CK1. Results obtained for the 10 different similarity templates (inlets **a**–**j**) along with their corresponding pdb codes are shown. Results from screens following the two distinct approaches with respect to redundancy are distinguished for each system.

The screening efficacy was acceptable in 9 out of 10 screens of the ligand-based approach (Figure 2). In one system (screen based on 4TW9 query) the performance was impressively poor (Figure 2i), actually affording “anti-enrichment” values. A possible reason for this result might be the extended structure of this particularly large CK1 inhibitor (MW of 586 Da), the dimensions of which are, however, not beyond limits reasonable for justifying its avoidance as a template in advance of the screen. Regarding the 9 successful screens, the major observation was again the clear correlation between redundancy treatment and performance. In this case, the effect on the overall enrichment scores was dramatic. A systematic decrease in the ROC AUC values was observed for the shift from the “redundant” to “no duplicates” approach, with a drop in performance in the range of 20–30% and in some cases up to 34% (from 0.929 to 0.585 in the 4G17-based query, Figure 2d). Such a sharp drop resulted in screens where remarkably poor enrichment was obtained (screen based on 2CHL query, ROC AUC of 0.543). Yet, the same shift toward the “no duplicates” approach smoothened the “anti-enrichment” of the 4TW9-based screen into a poor but reasonable result (ROC AUC from 0.220 to 0.515, RIE and BEDROC from 0 to 0.452 and 0.026, respectively; Figure 2i). An additional observation was that the inconsistency trends observed in the structure-based screens with respect to the RIE and BEDROC metrics as compared to their corresponding ROC AUC values were, quite interestingly, reproduced in this case as well. Indeed, in poor agreement with ROC AUC shifts for the transition from the “redundant” to the “no duplicates” screens, the early enrichment scores showed an improvement in several screens of the ligand-based approach, which was considerable in specific instances (RIE from 0.018 to 0.520 and from 0.537 to 1.773, BEDROC from 0.005 to 0.072 and from 0.005 to 0.102; Figure 2a,j and Figure 2f,j, respectively).

### 2.6. Combined Screening Approach

**Figure 3:**
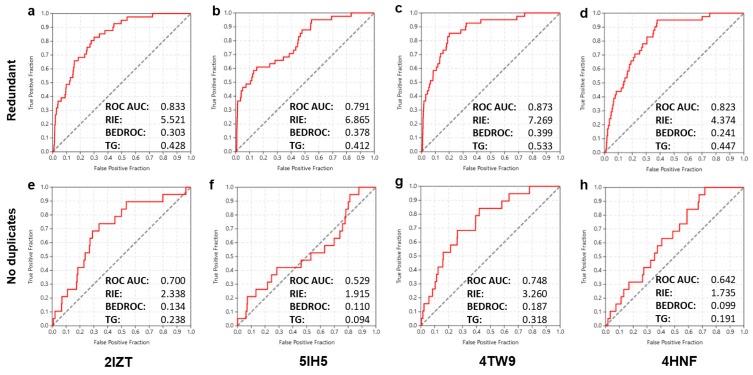
The ROC curves and key enrichment metrics obtained for the final combined screening effort of the NCI Diversity Set-II against CK1. Results obtained for the 3 high-performance (2IZT, 5IH5 and 4HNF) and 1 low-performance (4TW9) templates along with their corresponding pdb codes are shown (inlets **a**–**h**). In the first (**a**–**d**) and second (**e**–**h**) lines, data for screens following the two distinct approaches with respect to redundancy are depicted.

As a final step, an assessment was performed on whether insight derived previously by sequential implementation of the two separate methodologies could be exploited prospectively for further enhancing overall screening performance. In other words, this combined approach would assist in determining whether the protein structure of a successful ligand-based query could offer a suitable template for equally successful structure-based screening as well. Stepwise implementation of the faster ligand-based method in advance of the generally slower structure-based screen was deemed as a reasonable approach for the order of combining the two techniques (mean calculation times for screening a preprocessed enumerated file of the NCI Diversity Set-II: ~1 h for ROCS, ~4 h for Glide-SP, ~70 h for Glide-XP on a typical i7 processor). To this end, the corresponding protein structures of three queries from the ligand-based approach that afforded the highest performance (pdb codes: 2IZT, 5IH5, 4HNF) and one which afforded the lowest (4TW9) were utilized as docking templates for independent structure-based screening. The pre-optimized docking settings with respect to VdW scaling and screening accuracy level were used as determined at the first step of the study. Results and corresponding metrics are summarized in Figure 3 and show that although the two methods are orthogonal, the attempted combined implementation was not successful in advancing enrichment significantly. The best results in this final round of structure-based screening were, quite surprisingly, obtained by the template structure affording the poorest performance in the ligand-based screen (4TW9, Figure 3c,g). The ROC AUC and early enrichment metrics were reasonably high and, notably, slightly improved in comparison to the corresponding scores of the best performing systems in the initial docking screen (Figure 1c,h). On the other hand, the three templates originating from the successful ligand-based screening cases resulted in lower enrichments and in one case, (5IH5, Figure 3f) in a markedly low ROC AUC score close to randomness (0.529). An additional observation in this final combined screening effort was the rather anticipated decrease in screening performance by the shift from the “redundant” to the “no duplicates” approach, which was evident in all screens performed in this study.

### 2.7. Overview of Ranking Success and Practical Considerations

The enrichment results and metrics described in the previous sections represent an integrated and, in a sense, macroscopic evaluation for the capacity of each in silico method to rank order the 19 compounds that showed measurable CK1 inhibition at the top of the corresponding screening score-defined lists. The metrics used in such benchmarking assessments, however useful, they are themselves not sufficiently narrative, and they actually become meaningful only upon comparison with other similar screening efforts (assessments based on different virtual screening algorithms but which follow a retrospective approach with fully consistent experimentally derived datasets). This kind of comparative analysis is a perfectly acceptable practice in the literature [4,36] and is used throughout the present study. Yet, in an effort to provide a more practical aspect of the comparative work performed herein, we focus on the 4 most interesting compounds in terms of CK1 inhibitory potency (Table 1) and provide a summary of their ranks achieved by the most successful screen of each approach. In Table 2, the fraction of the compounds one would have to screen in reality in vitro for recovering all 4 actives is shown as a percentage of the total collection (screening percentage metric, see [40]). On the basis of this metric, the actual performance of each methodology varies depending on the fraction of actives one wishes to ensure. In case a 100% recovery of all 4 potent inhibitors is aimed, then the ligand-based method shows the best performance (45.82%), which nonetheless represents a poor overall enrichment. However, in a realistic situation, recovery of a fraction of actives that is considerably larger that the hit rate of randomness (in this specific case 0.29%) can be considered as a success. In this sense, recovering 50% of actives (i.e., 2 compounds or the second lowest percentage in Table 2) represents a substantial increase in enrichment as compared to random compound selection and, in this case, structure-based screening performs far better (screening percentage 1.76%, 28-fold enrichment) than both ligand-based or combined screening (screening percentages 9.80% and 10.79%, respectively, and approximately 5-fold enrichment). Interestingly, the most active compound **1** demonstrates high screening percentages in all methods and this notable tendency of NSC45572 to rank low possibly represents an inherent structural feature that enhances promiscuity of this compound to systematically act as a false negative. A likely explanation could be that **1** carries only one amine and one carbonyl group for H-bonding and stabilizes inside the kinase through extended stacking interactions. Consequently, given that many scoring schemes fail to model accurately the various aromatic-alkyl/aromatic-aromatic interactions, this might lead to a systematic underestimation of the binding affinity for compound **1**.

A major notion also related to the practical aspects of true actives recovery is that when a real prospective screening endeavor is planned, in contrast to a retrospective evaluation of already assessed bioactivities, then the “redundant” approach collapses into the “no duplicates” by the fact that each molecule can either be selected for experimental evaluation or discarded only once. The observed variations and the pronounced effect that this differentiation had on enrichment metrics can be attributed to reasons related to the uneven expansion of the compound collection upon enumeration. A typical cause, observed throughout in this study as well, is existence of molecules that due to their size are non-binders (compounds that afford bad screening scores due to steric clashes upon docking or a complicated molecular shape). Such molecules do happen to have many different possible tautomeric states or isomers exactly because of their size (large number of nitrogens or chiral atoms etc.). In a screening effort, those molecules will accumulate to the bottom of the ranked list but they will populate it with a large number of possible states, thus creating an artificial increase in all enrichment metrics based on the “redundant” approach. In this sense, we underline the ambiguous performance metrics that such an approach may introduce in a retrospective evaluation and we draw the attention to the careful usage of it.

### 2.8. Cell-Based Assessment of p53-Modulating Capacity of NSC45572

**Figure 4:**
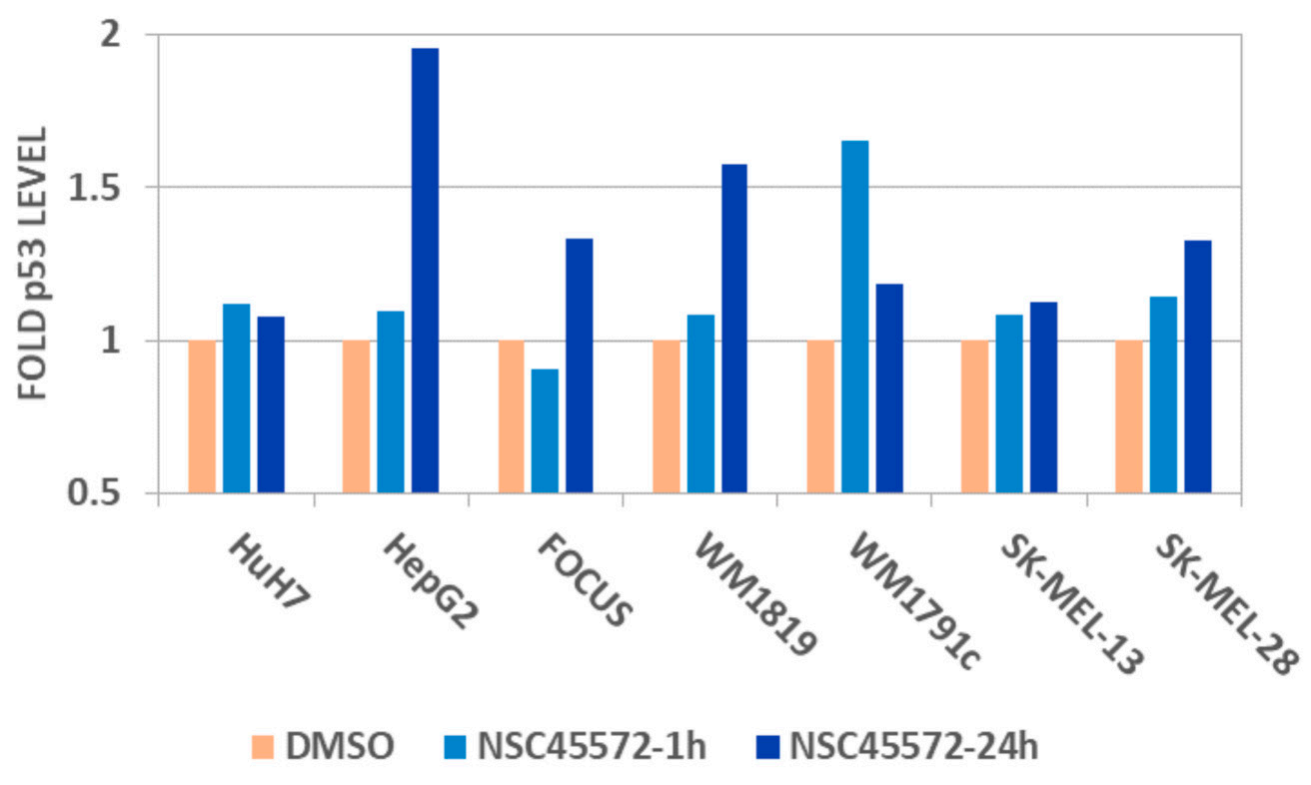
A graph showing the effect on p53 levels after treatment with 10 μM of compound **1** (NSC45572) in a number of malignant cell lines (hepatocellular carcinoma: HuH7, HepG2, FOCUS; melanoma: WM1819, WM1791c, SK-MEL-13, SK-MEL-28) at two time-points (1 and 24 h). A systematic and in several cases (HepG2 cells) significant increase of p53 level is observed in most cell lines, especially after 24 h of treatment, with the exception of HuH7 cells that carry a mutant *TP53* gene and the SK-MEL-13 line where the effect is limited. DMSO: dimethyl sulphoxide.

As already discussed, one of the most interesting aspects of CK1 activity is regulation of the tumor suppressor p53. CK1 stabilizes p53 through two distinct mechanisms; first, it phosphorylates p53 mainly at residues Thr18 and Ser20, thus leading to inhibition of its negative interaction with MDM2 which is responsible for p53 turnover and second, it also directly phosphorylates MDM2 leading to its degradation [41,42,43]. The discovery of compound **1** as a potent CK1 inhibitor prompted us to investigate its capacity to modulate the endogenous levels of p53 in cells. To this end, we used a panel of 7 pathological cell lines with a variety of basal p53 expression profiles (hepatocellular carcinoma cells HepG2, HuH7, FOCUS, melanoma cells WM1819, WM1791c, SK-MEL-28, SK-MEL-13) and monitored the protein levels at two time points (1 and 24 h) after treatment with 10 μM of **1** using the Luminex bead-based ELISA assay platform (ProtATonce Ltd., Ag. Paraskevi, Greece) [44]. Prior to p53 measurements, possible antiproliferative effects of the studied compound were assessed utilizing a Resazurin cell viability assay. It was shown that **1** had no measurable effects on cell proliferation at 10 μM over most of the studied cell lines, with the exception of a slight effect observed after 24 h treatment in the low basal p53 expression melanoma cell lines WM1819 and WM1791c (cell proliferation reduced to 77% and 85% of control, respectively). The subsequent Luminex measurements showed that a significant increase of p53 was observed in almost all cell lines upon treatment with 10 μM of **1** (Figure 4). The highest increase in the protein levels ranged from 1.3-fold to almost 2-fold and were determined after 24 h of treatment in the hepatocellular carcinoma lines HepG2 and FOCUS and the melanoma lines WM1819 and SK-MEL-28. The only exception was the slight decrease of p53 levels after 1 h of treatment in the FOCUS line, which was however fully reversed at 24 h, resulting to a final 1.3-fold increase. Notably, the effect on the Huh7 line carrying a mutant *TP53* gene was limited, as was the overall effect in the melanoma SK-MEL-13 cells.

### 2.9. Docking of NSC45572 in the CK1 Active Site

**Figure 5:**
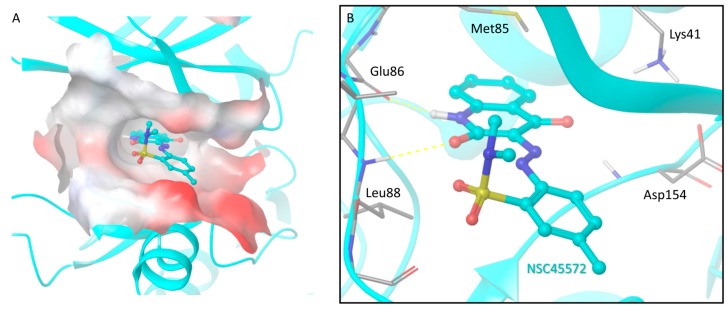
The proposed binding mode of compound **1** (NSC45572) inside the CK1 binding pocket. The pocket is depicted as a molecular surface colored according to the protein electrostatic potential (inlet **A**). The inhibitor binds the kinase hinge by adopting a type-I geometry and is stabilized by two hydrogen bonds (shown as dashed lines) formed between its lactam system and two backbone sites of residues Glu86 and Leu88, while the sulphonamide group orients in a perpendicular conformation towards the binding site periphery, thus avoiding any serious steric clashes with the protein walls (inlet **B**).

Finally, to gain insight to the interactions between the cell-active CK1 inhibitor **1** and its target, an exhaustive docking analysis was performed by implementing the induced-fit docking algorithm (Schrodinger Inc.) [45,46,47]. The proposed binding mode of the ligand resembles that of a typical type-I inhibitor geometry (Figure 5) where the aromatic system of **1** is tightly packed inside the kinase binding pocket through hydrophobic and stacking interactions, while two hydrogen bonds formed between the lactam ring of the ligand and corresponding backbone groups of the kinase hinge anchor the inhibitor into the ATP-bind pocket of CK1.

## 3. Materials and Methods

### 3.1. Protein Kinase Assays

Sodium orthovanadate, egtazic acid (EGTA), ethylenediaminetetraacetic acid (EDTA), 3-Morpholinopropane-1-sulfonic acid (Mops), β-glycerophosphate, phenylphosphate, sodium fluoride, dithiothreitol (DTT), glutathione-agarose, glutathione, bovine serum albumin (BSA), nitrophenylphosphate, leupeptin, aprotinin, pepstatin, soybean trypsin inhibitor, benzamidine, and histone H1 (type III-S) were obtained from Sigma Chemicals. [γ-^33^P]-ATP was obtained from Amersham. The CK-S peptide (RRKHAAIGpSAYSITA) (pS stands for phosphorylated serine) was purchased from Millegen (Labège, France), and the GS-1 peptide (YRRAAVPPSPSLSRHSSPHQpSEDEEE) was obtained from the Gen-Script Corporation (Piscataway Township, NJ, USA). Buffer A: 10 mM MgCl_2_, 1 mM EGTA, 1 mM DTT, 25 mM Tris-HCl pH 7.5, 50 μg heparin/mL. Buffer C: 60 mM β-glycerophosphate, 15 mM *p*-nitrophenylphosphate, 25 mM Mops (pH 7.2), 5 mM EGTA, 15 mM MgCl_2_, 1 mM DTT, 1 mM sodium vanadate, and 1 mM phenylphosphate. Kinase activities were assayed in buffer A or C, at 30 °C, at a final ATP concentration of 15 μM. Blank values were subtracted and activities expressed in % of the maximal activity, i.e., in the absence of inhibitors. Controls were performed with appropriate dilutions of dimethyl sulphoxide (DMSO). CDK5/p25 (human, recombinant) was prepared as previously described [48]. Its kinase activity was assayed in buffer C, with 1 mg histone H1/mL, in the presence of 15 μM [γ-^33^P] ATP (3000 Ci/mmol; 10 mCi/mL) in a final volume of 30 μL. After 30 minutes of incubation at 30 °C, 25-μL aliquots of supernatant were spotted onto 2.5 cm × 3 cm pieces of Whatman P81 phosphocellulose paper, and, 20 s later, the filters were washed five times (for at least 5 min each time) in a solution of 10 mL of phosphoric acid/liter of water. The wet filters were counted in the presence of 1 mL of ACS (Amersham, UK) scintillation fluid. DYRK1A (human, recombinant, expressed in *Escherichia coli* as GST fusion proteins) was assayed as described for CDK5/p25 with 1 μg of RS peptide (GRSRSRSRSRSR) as a substrate. GSK-3α/β (porcine brain, native) was assayed as described for CDK5 but in buffer A and using GS-1, a GSK-3-specific substrate [49]. CK1 (porcine brain, native) was assayed as described for CDK1 but using 0.67 μg of CKS peptide (RRKHAAIGpSAYSITA), a CK1-specific substrate [50].

### 3.2. Cell Cultures

The hepatocellular carcinoma cells of HepG2, HuH7, and FOCUS, and melanoma SK-MEL-28, SK-MEL-13, WM1819, and WM1791c were provided by ProtATonce Ltd. Cell lines were cultured in RPMI medium (Thermo Fischer Scientific, Waltham, MA, USA, 11875093) supplemented with 10% fetal bovine serum (Biosera, Nuaille, France, FB1001) and 1% penicillin/streptomycin (Thermo Fischer Scientific, 15140148) in a 37 °C, 5% CO_2_, humidified incubator. Cells were seeded in 96-well plates (Corning Inc., Corning, NY, USA, 3599) at the optimum seeding densities for each cell line, and after 24 h they were treated with the test compound in 0.1% DMSO or DMSO for 1 h and 24 h. After the treatment cells were lysed using lysis buffer optimized for phosphoproteomic measurements (ProtaVio Ltd., Stevenage, UK) along with protease/phosphatase inhibitor mix (ProtaVio Ltd.) and phenylmethanesulfonyl fluoride (PMSF; SIGMA, P4626). A Micro BCA™ Protein Assay Kit (Thermo Fisher Scientific, 23235) was used to measure the protein content of the lysates in order to adjust their concentrations to the same levels across samples before ELISA measurement. BSA was used for standard curve construction. Absorbance was measured at 562 nm using the Varioskan™ LUX multimode microplate reader (Thermo Fisher Scientific™).

### 3.3. Cell Viability Assay

To assess the potential antiproliferative effects, cell viability was assessed using the Resazurin cell viability assay (SantaCruz, Dallas, TX, USA, SC-206037). Resazurin viability assay is widely used in measuring cell proliferation and cytotoxicity. Resazurin is a non-fluorescent compound and is reduced by the intracellular nicotinamide adenine dinucleotide (NADH) of live cells to resorufin, which is highly fluorescent. In this study, resazurin sodium salt was initially diluted and stored in Dulbecco’s phosphate buffered saline pH 7.4 (Biosera, PM-B2092), and then further diluted in culture medium before every experiment. After the 1 and 24 h treatment, culture medium was replaced with the addition of 10 μg/mL Resazurin. Following 2-h incubation in a 37 °C, 5% CO_2_, humidified incubator, fluorescence was measured at wavelength Ex560 nm/Em590 nm using a Varioskan™ LUX multimode microplate reader (Thermo Fisher Scientific™). Cell viability was expressed as a percentage of treated to untreated cells.

### 3.4. xMAP Assays

The xMAP assays were performed on a Luminex-200 platform (Luminex, Austin, TX, USA) using custom phosphoprotein antibody-coupled beads (ProtATonce, Athens, Greece). A custom multiplex was used to determine in cell lysates the levels of test phosphoprotein p53. The fold change of the signals relative to the unstimulated state was calculated.

### 3.5. Preparation of the Compound Collection, Virtual Screening and Induced-Fit Docking

Prior to virtual screening, the NCI Diversity Set-II compounds were enumerated in terms of correct protonation state, tautomerization, stereoisomer representation, and conformer generation (for ROCS only) using tools provided by the corresponding screening packages (LigPrep module for Glide and Filter, Quacpac, Omega for ROCS). Omega was used with a limit of 200 conformers generated for each molecule. Concerning Glide, prior to grid generation, all protein structures were prepared by using the Protein Preparation module (Schrodinger Inc.) in terms of correct bond order, with addition of missing hydrogens and removal of crystallographic waters, and then a restrained energy minimization was applied. All grids were prepared using a scaling factor of 0.8 for the Van der Waals radii of protein atoms. Different docking calculations were performed for each grid by using a ligand Van der Waals scaling factor of 0.8 or 1.0 and sampling was performed by either SP or XP algorithms using the default GlideScore scoring function. With respect to ROCS screening, the implicit Dean–Mills force-field and the Tanimoto combo scoring scheme were used. The induced-fit docking (IFD) protocol as implemented in the Schrodinger suite (Schrodinger Inc.) was used for re-docking NSC45572 to the active site of CK1 at a high-accuracy level [45,46,47]. The docking grid was based on the 2IZR crustal structure and was prepared using the ProtPrep module as discussed above.

### 3.6. Enrichment Quantification

For all enrichment and screening efficacy calculations, the threshold for discriminating compounds between active and inactive was a residual kinase activity of 40% as determined in the experimental enzyme inhibition measurements. All ranked lists were preprocessed in Microsoft Excel (Microsoft Inc., Albuquerque, NM, USA) and analyzed by the Screening Explorer tool available on-line [37]. Details on the formalism of enrichment metrics used in this study can be found in [4,36]. For determination of the BEDROC and RIE metrics, an α value of 20 was used.

## 4. Conclusions

With the objective of discovering novel scaffolds of high structural originality showing activity against proteins of potential therapeutic interest, efficient navigation across the vast chemodiversity available inside the various academic and commercial compound collections is a prerequisite for enhancing hit rate and rationalizing the whole process of primary hit validation. In this endeavor, integration of theoretical in silico methodologies with experimental in vitro high-throughput screening approaches can have a decisive contribution towards accelerating the progress and enhancing the success rate of screening campaigns. In this study, by using representative tools and software, we successfully identified a potent and selective CK1 inhibitor, and we showed that of the two main methods available in terms of in silico screening algorithms, the docking-scoring structure-based approach performs slightly better than the ligand-based screening, however both can sustain a fairly successful screening campaign either on their own or by a simple combination. This notion is well reflected in the mean values obtained for the most descriptive metric in terms of overall enrichment, ROC AUC, which reached 0.714 for the initial structure-based screen, 0.613 for the subsequent ligand-based counterpart, and 0.654 for the final combined screening effort.

However, results showed that the major variation in the apparent efficacy of the benchmarked methods did not originate from the selection of screening method but from treatment of parameters that, to our view, are usually ignored upon setup of such screening endeavors. In accordance with similar methodological studies [3,4,6], we showed that by taking into consideration different screening templates, either crystal structures or known ligands in their bioactive conformation depending on the screening approach followed, we could achieve a variety of enrichment metrics that ranged between high, acceptable, but also exceedingly poor performance. Such a typical case was the resulting enrichment obtained by the 4TW9 template in the ligand-based screening effort. Yet, no straightforward algorithm for selection of the most suitable template in each case exists and performing a large number of screens using different crystal structures or having access to an adequately extensive training set are the only confident ways for rationally making such a choice.

Another critical parameter, the importance of which was clearly demonstrated by the benchmarking data presented herein, was treatment of redundancy in terms of the way all possible states of the screened molecules are conclusively considered in the ranking stage of the screen. The crucial effect of this factor on apparent performance is evident in the corresponding mean ROC AUC values that demonstrate a sharp but misleading increase to 0.804, 0.840, and 0.830 for the initial structure-based, the subsequent ligand-based, and the final combined screen, respectively. This effect shows a striking and actually unwanted delicacy in determinants influencing screening quality without being essential components of the main screening machinery, either structure-based or ligand-based. To our view, the treatment considering only the top ranked state of each compound (designated in this study as “no duplicates”) represents the most rational approach to dealing with redundancy. Moreover, it is preferred on the basis of its compatibility with prospective screening campaigns and not only retrospective studies, although it systematically affords lower enrichment metrics and poorer apparent screening performance.

The questionable delicacy of the assessed screening methods in terms of performance was additionally reflected on the final attempt of this study to combine insight derived by subsequent orthogonal screens and achieve higher enrichment compared to the individual techniques. The combined approach showed fairly good performance in terms of the ROC AUC value that was not considerably improved when compared to the individual screening methods. This combined attempt however, afforded a poor result in terms of the percentage that had to be screened for recovery of the most potent CK1 inhibitors. Moreover, the fact that in this attempt the best template for structure-based screening was proven to be the one affording the lowest performance in the ligand-based effort clearly shows that in cases where no cross-validation measures such as a training set have been secured, then a screen is in part subject to serendipitous attitude and this has to be taken into account when planning screening campaigns.

Finally, to respect to the discovered CK1 inhibitor, we consider that it represents a highly original and, in this respect, promising starting point for development of non-toxic compounds with the potential to selectively modulate the endogenous levels of the key factor for genomic stability, p53. The detailed study of its cellular effects is planned, with a more extensive analysis of its complete regulatory profile using a proteomics approach. This study is anticipated to shed light to the various mechanisms of its cellular action and assist in determining whether its further synthetic development into a series of analogues would be worthy.

## Figures and Tables

**Table 1:** The structures of the four NCI Diversity Set-II compounds affording the best results in the kinase inhibition assay towards CK1δ/ε, along with their residual enzyme activities at 10 μM and their selectivity against three related protein kinases.

Compound Code	Structure	Residual Kinase Activity			
		CK1δ/ε	GSK3α/β	CDK5/p25	DYRK1α
NSC45572 (**1**)	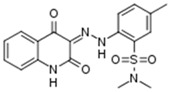	**7%**	82%	77%	94%
NSC252777 (**2**)	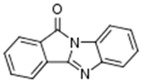	**18%**	96%	98%	72%
NSC71866 (**3**)	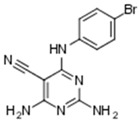	**14%**	74%	**25%**	72%
NSC105827 (**4**)	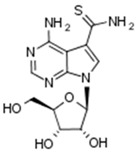	**20%**	33%	**7%**	**12%**

CK: casein kinase; GSK: glycogen synthase kinase; CDK: cyclin-dependent kinase; DYRK: dual specificity tyrosine-phosphorylation-regulated kinase.

**Table 2:** The screening percentage or fraction of the NCI Diversity Set-II collection needed to be experimentally evaluated for recovering each of the 4 most potent CK1 inhibitors. Values were determined on the basis of ranking achieved by the optimally performing structure-based, ligand-based, and combined screening methods, as they are individually described in Section 2.4, Section 2.5 and Section 2.6, respectively. More specifically, all values have been calculated by dividing the rank of each active compound by the total number of entries in each of the described screening calculations and subsequently determining the corresponding percentages.

Active Compound	Structure-Based Screening	Ligand-Based Screening	Combined Screening
NSC45572	82.52%	45.82%	78.41%
NSC252777	7.48%	9.80%	10.79%
NSC71866	1.76%	1.21%	40.01%
NSC105827	0.81%	19.67%	0.66%

## References

[1] Shoichet B.K. (2004). Virtual screening of chemical libraries. Nature.

[2] Zhao W., Hevener K.E., White S.W., Lee R.E., Boyett J.M. (2009). A statistical framework to evaluate virtual screening. BMC Bioinform..

[3] Chaput L., Martinez-Sanz J., Saettel N., Mouawad L. (2016). Benchmark of four popular virtual screening programs: Construction of the active/decoy dataset remains a major determinant of measured performance. J. Cheminform..

[4] Kirchmair J., Markt P., Distinto S., Wolber G., Langer T. (2008). Evaluation of the performance of 3D virtual screening protocols: RMSD comparisons, enrichment assessments, and decoy selection—What can we learn from earlier mistakes?. J. Comput. Aided Mol. Des..

[5] Mysinger M.M., Carchia M., Irwin J.J., Shoichet B.K. (2012). Directory of useful decoys, enhanced (DUD-E): Better ligands and decoys for better benchmarking. J. Med. Chem..

[6] Madhavi Sastry G., Adzhigirey M., Day T., Annabhimoju R., Sherman W. (2013). Protein and ligand preparation: parameters, protocols, and influence on virtual screening enrichments. J. Comput. Aided Mol. Des..

[7] Arkin M.R., Tang Y., Wells J.A. (2014). Small-molecule inhibitors of protein–protein interactions: Progressing toward the reality. Chem. Biol..

[8] Laraia L., McKenzie G., Spring D.R., Venkitaraman A.R., Huggins D.J. (2015). Overcoming chemical, biological, and computational challenges in the development of inhibitors targeting protein–protein interactions. Chem. Biol..

[9] Pearl L.H., Schierz A.C., Ward S.E., Al-Lazikani B., Pearl F.M.G. (2015). Therapeutic opportunities within the DNA damage response. Nat. Rev. Cancer.

[10] Jeggo P.A., Pearl L.H., Carr A.M. (2016). DNA repair, genome stability and cancer: A historical perspective. Nat. Rev. Cancer.

[11] Jackson S.P., Bartek J. (2009). The DNA-damage response in human biology and disease. Nature.

[12] Petrakis T.G., Komseli E.-S., Papaioannou M., Vougas K., Polyzos A., Myrianthopoulos V., Mikros E., Trougakos I.P., Thanos D., Branzei D. (2016). Exploring and exploiting the systemic effects of deregulated replication licensing. Semin. Cancer Biol..

[13] Zhang D., Wang H.-B., Brinkman K.L., Han S.-X., Xu B. (2012). Strategies for targeting the DNA damage response for cancer therapeutics. Chin. J. Cancer.

[14] Knippschild U., Gocht A., Wolff S., Huber N., Löhler J., Stöter M. (2005). The casein kinase 1 family: Participation in multiple cellular processes in eukaryotes. Cell. Signal..

[15] Knippschild U., Krüger M., Richter J., Xu P., García-Reyes B., Peifer C., Halekotte J., Bakulev V., Bischof J. (2014). The CK1 family: Contribution to cellular stress response and its role in carcinogenesis. Front. Oncol..

[16] Huart A.-S., MacLaine N.J., Meek D.W., Hupp T.R. (2009). CK1α plays a central role in mediating MDM2 control of p53 and E2F-1 protein stability. J. Biol. Chem..

[17] Meek D.W. (2015). Regulation of the p53 response and its relationship to cancer. Biochem. J..

[18] Vassilev L.T., Vu B.T., Graves B., Carvajal D., Podlaski F., Filipovic Z., Kong N., Kammlott U., Lukacs C., Klein C. (2004). In vivo activation of the p53 pathway by small-molecule antagonists of MDM2. Science.

[19] Greer Y.E., Gao B., Yang Y., Nussenzweig A., Rubin J.S. (2017). Lack of casein kinase 1 delta promotes genomic instability—The accumulation of DNA damage and down-regulation of checkpoint kinase 1. PLoS ONE.

[20] Peifer C., Abadleh M., Bischof J., Hauser D., Schattel V., Hirner H., Knippschild U., Laufer S. (2009). 3,4-Diaryl-isoxazoles and -imidazoles as potent dual inhibitors of p38α mitogen activated protein kinase and casein kinase 1δ. J. Med. Chem..

[21] Oumata N., Bettayeb K., Ferandin Y., Demange L., Lopez-Giral A., Goddard M.-L., Myrianthopoulos V., Mikros E., Flajolet M., Greengard P. (2008). Roscovitine-derived, dual-specificity inhibitors of cyclin-dependent kinases and casein kinases 1. J. Med. Chem..

[22] Rena G., Bain J., Elliott M., Cohen P. (2004). D4476, a cell-permeant inhibitor of CK1, suppresses the site-specific phosphorylation and nuclear exclusion of FOXO1a. EMBO Rep..

[23] Laco F., Low J.-L., Seow J., Woo T.L., Zhong Q., Seayad J., Liu Z., Wei H., Reuveny S., Elliott D.A. (2015). Cardiomyocyte differentiation of pluripotent stem cells with SB203580 analogues correlates with Wnt pathway CK1 inhibition independent of p38 MAPK signaling. J. Mol. Cell. Cardiol..

[24] Lee J.W., Hirota T., Peters E.C., Garcia M., Gonzalez R., Cho C.Y., Wu X., Schultz P.G., Kay S.A. (2011). A small molecule modulates circadian rhythms through phosphorylation of the period protein. Angew. Chem. Int. Ed..

[25] Walton K.M., Fisher K., Rubitski D., Marconi M., Meng Q.-J., Sládek M., Adams J., Bass M., Chandrasekaran R., Butler T. (2009). Selective inhibition of casein kinase 1ε minimally alters circadian clock period. J. Pharmacol. Exp. Ther..

[26] Badura L., Swanson T., Adamowicz W., Adams J., Cianfrogna J., Fisher K., Holland J., Kleiman R., Nelson F., Reynolds L. (2007). An inhibitor of casein kinase Iε induces phase delays in circadian rhythms under free-running and entrained conditions. J. Pharmacol. Exp. Ther..

[27] Myrianthopoulos V., Gaboriaud-Kolar N., Tallant C., Hall M.-L., Grigoriou S., Brownlee P.M., Fedorov O., Rogers C., Heidenreich D., Wanior M. (2016). Discovery and optimization of a selective ligand for the switch/sucrose nonfermenting-related bromodomains of polybromo protein-1 by the use of virtual screening and hydration analysis. J. Med. Chem..

[28] Myrianthopoulos V., Cartron P.F., Liutkevičiūtė Z., Klimašauskas S., Matulis D., Bronner C., Martinet N., Mikros E. (2016). Tandem virtual screening targeting the {SRA} domain of {UHRF1} identifies a novel chemical tool modulating {DNA} methylation. Eur. J. Med. Chem..

[29] Friesner R.A., Banks J.L., Murphy R.B., Halgren T.A., Klicic J.J., Mainz D.T., Repasky M.P., Knoll E.H., Shelley M., Perry J.K. (2004). Glide: A new approach for rapid, accurate docking and scoring. 1. Method and assessment of docking accuracy. J. Med. Chem..

[30] Halgren T.A., Murphy R.B., Friesner R.A., Beard H.S., Frye L.L., Pollard W.T., Banks J.L. (2004). Glide: A new approach for rapid, accurate docking and scoring. 2. enrichment factors in database screening. J. Med. Chem..

[31] Friesner R.A., Murphy R.B., Repasky M.P., Frye L.L., Greenwood J.R., Halgren T.A., Sanschagrin P.C., Mainz D.T. (2006). Extra precision glide: Docking and scoring incorporating a model of hydrophobic enclosure for protein−ligand complexes. J. Med. Chem..

[32] Hawkins P.C.D., Skillman A.G., Nicholls A. (2007). Comparison of shape-matching and docking as virtual screening tools. J. Med. Chem..

[33] Empereur-mot C., Guillemain H., Latouche A., Zagury J.-F., Viallon V., Montes M. (2015). Predictiveness curves in virtual screening. J. Cheminform..

[34] Feher M. (2006). Consensus scoring for protein–ligand interactions. Drug Discov. Today.

[35] Triballeau N., Acher F., Brabet I., Pin J.-P., Bertrand H.-O. (2005). Virtual screening workflow development guided by the “receiver operating characteristic” curve approach. Application to high-throughput docking on metabotropic glutamate receptor subtype 4. J. Med. Chem..

[36] Truchon J.-F., Bayly C.I. (2007). Evaluating virtual screening methods: Good and bad metrics for the “early recognition” problem. J. Chem. Inf. Model..

[37] Empereur-Mot C., Zagury J.-F., Montes M. (2016). Screening explorer–An interactive tool for the analysis of screening results. J. Chem. Inf. Model..

[38] Polychronopoulos P., Magiatis P., Skaltsounis A.-L., Myrianthopoulos V., Mikros E., Tarricone A., Musacchio A., Roe S.M., Pearl L., Leost M. (2004). Structural basis for the synthesis of indirubins as potent and selective inhibitors of glycogen synthase kinase-3 and cyclin-dependent kinases. J. Med. Chem..

[39] Huse M., Kuriyan J. (2002). The conformational plasticity of protein kinases. Cell.

[40] Chen H., Lyne P.D., Giordanetto F., Lovell T., Li J. (2006). On evaluating molecular-docking methods for pose prediction and enrichment factors. J. Chem. Inf. Model..

[41] Sakaguchi K., Saito S., Higashimoto Y., Roy S., Anderson C.W., Appella E. (2000). Damage-mediated phosphorylation of human p53 threonine 18 through a cascade mediated by a casein 1-like kinase: Effect on MDM2 binding. J. Biol. Chem..

[42] MacLaine N.J., Øster B., Bundgaard B., Fraser J.A., Buckner C., Lazo P.A., Meek D.W., Höllsberg P., Hupp T.R. (2008). A central role for CK1 in catalyzing phosphorylation of the p53 transactivation domain at serine 20 after HHV-6B viral infection. J. Biol. Chem..

[43] Inuzuka H., Tseng A., Gao D., Zhai B., Zhang Q., Shaik S., Wan L., Ang X.L., Mock C., Yin H. (2010). Phosphorylation by casein kinase I promotes the turnover of the MDM2 oncoprotein via the SCFβ-TRCP ubiquitin ligase. Cancer Cell.

[44] Alexopoulos L.G., Saez-Rodriguez J., Cosgrove B.D., Lauffenburger D.A., Sorger P.K. (2010). networks inferred from biochemical data reveal profound differences in toll-like receptor and inflammatory signaling between normal and transformed hepatocytes. Mol. Cell. Proteom. MCP.

[45] Sherman W., Day T., Jacobson M.P., Friesner R.A., Farid R. (2006). Novel procedure for modeling ligand/receptor induced fit effects. J. Med. Chem..

[46] Sherman W., Beard H.S., Farid R. (2006). Use of an induced fit receptor structure in virtual screening. Chem. Biol. Drug Des..

[47] Farid R., Day T., Friesner R.A., Pearlstein R.A. (2006). New insights about HERG blockade obtained from protein modeling, potential energy mapping, and docking studies. Bioorg. Med. Chem..

[48] Bettayeb K., Oumata N., Echalier A., Ferandin Y., Endicott J.A., Galons H., Meijer L. (2008). CR8, a potent and selective, roscovitine-derived inhibitor of cyclin-dependent kinases. Oncogene.

[49] Bach S., Knockaert M., Reinhardt J., Lozach O., Schmitt S., Baratte B., Koken M., Coburn S.P., Tang L., Jiang T. (2005). Roscovitine targets, protein kinases and pyridoxal kinase. J. Biol. Chem..

[50] Reinhardt J., Ferandin Y., Meijer L. (2007). Purification of CK1 by affinity chromatography on immobilised axin. Protein Expr. Purif..

